# 41 The Prevalence of Secondary Traumatic Stress in Burn Care Clinicians

**DOI:** 10.1093/jbcr/iraf019.041

**Published:** 2025-04-01

**Authors:** Emma Turner, Karen Kowalske, Elizabeth Salazar, Kimberly Roaten

**Affiliations:** University of Texas Southwestern Parkland Medical Center; University of Texas Southwestern Medical Center; University of Texas Southwestern Parkland Medical Center; University of Texas Southwestern Parkland Medical Center

## Abstract

**Introduction:**

While the understanding of secondary traumatic stress (STS) has grown over the past 30 years, there is a lack of research examining STS amongst burn care clinicians. Burn care clinicians’ experiences are unique due to the combination of often-traumatic mechanism of burn injuries, provision of repetitive and painful treatment, and continuing to provide life-long care. The current study aimed to determine the prevalence of secondary traumatic stress in a sample of burn care clinicians across all occupational roles.

**Methods:**

Participants completed a one-time survey including measures of STS (Secondary Traumatic Stress Scale [STSS]) and demographic/occupational information. The sample included 102 clinicians working in one of the largest civilian burn centers in the nation. All clinicians providing direct patient care were eligible to participate, including: advance practice provider; support services (case manager, respiratory therapist, social worker); chaplain; therapists (occupational, physical, speech therapists); attending physicians (PM&R, surgical); PM&R resident/fellow; registered nurse; surgical resident/fellow; wound care technician; other.

**Results:**

Recruitment occurred from 11/2/23 through 7/25/24 with a completion rate of 83.74%. The mean STSS total score among all clinicians was 37.24 (SD = 12.57), falling in the Mild STS range and just below the recommended cut-off value (38) for likely experiencing PTSD at a diagnostic level due to STS. Of the three STSS symptom subscales (Intrusion, Avoidance, Arousal), the Avoidance subscale had the highest mean score (M = 15.17, SD = 5.37). Of the seventeen STSS individual items, item #10 (“I thought about my work with my patients when I didn’t intend to”) had the highest mean severity rating (M = 3.01, SD = 1.23). There was not a significant difference in STSS total scores across occupational groups, χ² (9, n = 101) = 8.77, p =.46. Notably, the two occupations with the highest mean STSS total scores, in the Moderate STS range, were wound care technicians (M = 43.00, SD = 14.69) and registered nurses (M = 42.41, SD = 14.43). Excluding residents (N = 70), mean STSS total score was 38.04 (SD = 13.35), falling above the recommended cut-off value.

**Conclusions:**

While mean STSS total scores were in the Mild STS range for this sample, several occupations with potentially higher rates of secondary exposure to the traumatic experiences of burn patients did have mean scores in the Moderate/clinically significant range. Burn clinician STS prevalence rates are comparable to similar healthcare occupational roles.

**Applicability of Research to Practice:**

These findings have implications regarding the mental and physical health of burn care clinicians but also the potential impact on patient care and the hospital system. Research should focus efforts towards prevention and intervention in this setting.

**Funding for the Study:**

N/A

